# EMR-Based Interventions on HPV Vaccination Initiation, Completion, and Receiving the Next Dose: A Meta-Analytic Review

**DOI:** 10.3390/vaccines12070739

**Published:** 2024-07-03

**Authors:** Nutthaporn Chandeying, Therdpong Thongseiratch

**Affiliations:** 1 Department of Obstetrics and Gynecology, Faculty of Medicine Vajira Hospital, Navamindradhiraj University, Bangkok 10300, Thailand; nutthaporn027@gmail.com; 2 Department of Pediatrics, Faculty of Medicine, Prince of Songkla University, Songkhla 90100, Thailand

**Keywords:** human papillomavirus vaccination, electronic medical records, vaccination uptake, systematic review, meta-analysis

## Abstract

Despite the acknowledged importance of Human Papillomavirus (HPV) vaccination in reducing HPV-related diseases, the influence of electronic medical records (EMR) on HPV vaccination uptake (HVU) remains underexplored. This study aimed to evaluate the efficacy of EMR-based interventions on HVU. A systematic review and meta-analysis of randomized controlled trials were performed, focusing on studies that primarily used EMR-based interventions to measure initiation rates, completion rates, and receipt of the next required vaccine dose. Subgroup analyses were conducted to assess the differential effects of supplementary strategies, provider feedback, and parental education or reminders on these outcomes. The results of the comprehensive analysis provided robust evidence for the significant role of EMR interventions, demonstrating an average increase of 4.7% in vaccine initiation, 6.6% in vaccine completion, and 7.2% in receipt of the next HPV vaccine dose. Additionally, the subgroup analyses indicated that provider feedback and parental education could further enhance the effectiveness of EMR-based interventions. These findings support the broader adoption of digital health technologies in vaccination programs, offering vital insights for healthcare providers, policymakers, and researchers, and emphasizing the need for continued technological innovation to improve public health outcomes.

## 1. Introduction

The Human Papillomavirus (HPV) is a significant global public health concern. It is the primary cause of cervical cancer and contributes to several other malignancies, including oropharyngeal, anal, penile, vulvar, and vaginal cancers [1]. The development and widespread implementation of HPV vaccination programs represent a critical step in preventive health, aiming to reduce the incidence of these HPV-related diseases [2]. Despite the availability and proven efficacy of the HPV vaccine, vaccination rates remain suboptimal globally. According to the World Health Organization (WHO), many countries report HPV vaccination coverage levels of less than 50%, far below the levels needed to achieve herd immunity and make a substantial impact on public health [1,2,3,4]. The low uptake and completion rates of the vaccine significantly limit its potential benefits, underscoring the urgent need for innovative strategies to improve HPV vaccination uptake (HVU) [5].

The widespread implementation of electronic medical record (EMR) systems has the potential to revolutionize the delivery of healthcare services and improve patient outcomes [6,7]. These digital systems enable the efficient management of patient care by integrating functionalities such as patient reminders, tracking vaccination statuses, and providing timely interventions to enhance coverage rates [8]. Consequently, EMR-based interventions have emerged as a promising strategy to address the issue of suboptimal HVU, offering a technological approach to boost vaccination rates [9]. The integration of EMR systems in healthcare settings can streamline healthcare delivery, reduce missed opportunities for vaccination, and ensure that patients receive timely and complete vaccination series.

Healthcare providers play a crucial role in influencing HPV vaccination uptake. Studies consistently identify provider recommendations as one of the key determinants of patients’ vaccination decisions [10,11,12]. Various provider-targeted interventions, such as training, reminders, and feedback, have demonstrated efficacy in improving HVU. For instance, provider-based interventions have been shown to increase vaccination initiation rates by approximately 3.7% [13]. These findings highlight the significant impact that healthcare providers can have on enhancing vaccination uptake through traditional methods. However, with the advent of digital healthcare services, there is an opportunity to integrate these provider-targeted interventions into EMR systems, further streamlining healthcare delivery and reducing the likelihood of missed vaccination opportunities [13,14].

Existing systematic reviews have primarily focused on provider-targeted and community-based interventions, providing limited insights into the role of EMR-based strategies [13,14,15,16]. As EMRs become increasingly prevalent in healthcare settings, it is essential to assess their potential impact on vaccination coverage. Understanding the efficacy of EMR-based interventions in improving HVU can inform healthcare providers and policymakers about the best practices for integrating digital health technologies into vaccination programs.

This meta-analysis aims to fill the existing knowledge gap by evaluating the impact of EMR-based interventions on improving HVU. Specifically, we sought to analyze the effects of these interventions on three key dimensions of HVU: initiation, completion, and administration of the next required vaccine dose. Additionally, we conducted a comparative study to assess the added benefits of two supplementary intervention methods alongside standard EMR-based interventions. The first supplementary strategy involves provider feedback related to HPV vaccine prescription, while the second strategy focuses on the active engagement of parents. This engagement can be achieved through the provision of pre-doctor-visit educational materials at the clinic or by using EMR-generated reminders or links to patients’ electronic health records. Our ultimate goal is to determine which of these additional strategies, if any, is more effective in enhancing HVU. By exploring the potential of EMRs, this study aims to provide valuable guidance for healthcare providers and establish a strong foundation for future research in this domain.

## 2. Materials and Methods

The systematic review protocol was registered in the PROSPERO database (CRD42023389004). The methodology and presentation of the findings adhered to the Preferred Reporting Items for Systematic Reviews and Meta-Analyses (PRISMA) guidelines 2020 [17] and the Cochrane Handbook for Systematic Reviews of Interventions [18].

### 2.1. Search Strategy

A systematic literature search was conducted on PubMed, PsycInfo, Web of Science, and the Cochrane Central Register of Controlled Trials in June 2023. The search strategy was designed to capture relevant studies published from 2006, the year of the HPV vaccine launch, to June 2023. Keywords related to EMR-based interventions, HPV vaccines, and vaccination uptake were used to ensure comprehensive coverage of the relevant literature. To illustrate the search strategy, below is the full example used for PubMed:

(“Electronic Health Records”[Mesh] OR “Medical Records Systems, Computerized”[Mesh] OR “electronic medical record”[tiab] OR “EMR”[tiab] OR “electronic health record”[tiab] OR “EHR”[tiab])
AND
(“Papillomavirus Vaccines”[Mesh] OR “HPV vaccine”[tiab] OR “Human Papillomavirus vaccine”[tiab] OR “HPV vaccination”[tiab])
AND
(“Vaccination Coverage”[Mesh] OR “vaccination uptake”[tiab] OR “vaccine uptake”[tiab] OR “vaccination rate”[tiab] OR “vaccine rate”[tiab] OR “vaccination completion”[tiab] OR “vaccine completion”[tiab] OR “vaccination initiation”[tiab] OR “vaccine initiation”[tiab] OR “receipt of next dose”[tiab])

The search terms were combined using Boolean operators to ensure that all relevant studies were captured. The same search terms were adapted for use on PsycInfo, Web of Science, and the Cochrane Central Register of Controlled Trials.

### 2.2. Eligibility Criteria

Peer-reviewed articles in English that fulfilled the PICOS strategy were included.

Population (P): The study population included children, adolescents, and young adults aged 9–26 years eligible for the WHO-recommended HPV vaccine, along with their parents or healthcare providers.

Intervention (I): EMR-based interventions were used to remind healthcare providers about the HPV vaccination, specifically aiming to promote HVU.

Comparison (C): We included studies with control groups, defined as either ‘usual condition’ or ‘alternative control’. The ‘usual condition’ refers to standard practices in place, such as routine HPV vaccination procedures without additional interventions. The ‘alternative control’ involves different interventions, like manual follow-ups or educational methods, serving as comparison points. Studies solely focusing on EMR-based interventions without a comparative control group, such as non-inferiority trials, were excluded to ensure a clear assessment of the intervention’s effectiveness.

Outcome (O): The outcome measures in our study focused on the impact of EMR-based interventions on various aspects of HVU, specifically looking at vaccination initiation, completion, and receipt of subsequent vaccine doses. ‘Initiation rates’ refer to the proportion of individuals who have received at least one dose of the HPV vaccine, signifying the start of the vaccination process. ‘Completion rates’ are defined as the percentage of individuals who have received all the recommended doses of the HPV vaccine, indicating full adherence to the vaccination schedule. Additionally, we evaluated the continuation of the HPV vaccine schedule through the ‘next required dose outcome,’ which measures the timely receipt of subsequent vaccine doses as per the recommended schedule. Our analysis considered both self-reported and provider-verified vaccination statuses as valid indicators of vaccination coverage. To maintain a focus on actual vaccination behavior, studies that only provided information on the knowledge, attitudes, or intentions regarding HPV vaccination without actual vaccination data were excluded from our analysis.

Study design (S): Only randomized controlled trials (RCTs) were considered.

### 2.3. Study Selection

The reference lists of relevant systematic reviews and primary studies were examined. NC and TT independently screened the titles and abstracts of the retrieved reports in Rayyan to identify potentially eligible studies. The full texts of these studies were then assessed for eligibility by a research assistant, NC, and TT.

### 2.4. Data Extraction

Information on the general study characteristics, intervention characteristics, sample characteristics, and data for calculating effect sizes were extracted for each study. Intention-to-treat data were used in studies that reported both intention-to-treat and per-protocol analyses. The sample sizes for cluster sampling studies were reduced using the reported design effect and the intracluster correlation coefficient [19]. All data were organized in Microsoft Excel and coded by a research assistant, NC, and TT. The authors were contacted if the data could not be retrieved from the publication.

### 2.5. Quality Assessment

TT and NC used the Cochrane’s Risk of Bias 2.0 tool independently to assess the risk of bias in the included RCTs and their respective protocols and trial registry records. Publication bias was assessed by visually examining the funnel plot of the main outcome [17].

### 2.6. Data Synthesis and Analysis

The difference in HVU between the post-intervention and control groups was quantified using the relative effect estimate. This measure provides a comparative assessment of risk differences in vaccination rates. Given the heterogeneity among the trials, we employed a random-effects model for all analyses [18]. In multi-armed trials that reported more than one comparison, the sample size was divided to avoid power inflation. The relative effect estimates were log-transformed, combined using random-effects meta-analysis, and exponentiated to produce a pooled relative effect estimate. The degree of statistical heterogeneity among studies was quantified using I^2^ statistics. An additional subgroup analysis was conducted to compare the effect sizes of the interventions that included additional provider feedback and parental education or reminders. The aim was to identify whether these added strategies significantly affected the HVU compared with interventions that did not include these additional components. All analyses, including subgroup analyses, were conducted using the Comprehensive Meta-Analysis Software 3.0 [20].

## 3. Results

**Figure 1:**
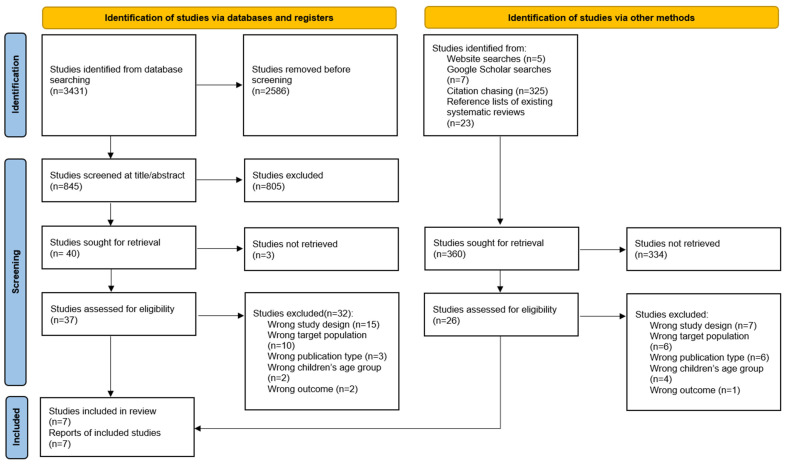
PRISMA 2020 flow diagram.

We identified 3431 articles during the initial database search. Seven RCTs were included in the review after applying the inclusion and exclusion criteria. The literature selection process is illustrated in Figure 1. The study characteristics are summarized in Table 1.

### 3.1. Characteristics of Included Studies

This systematic review included seven RCTs that assessed the effectiveness of EMR-based interventions on HPV vaccination uptake. These studies varied in sample size, geographic location, study population, and intervention type. All studies were conducted in the United States, and the sample sizes ranged from 648 to 22,486 participants. The age of the participants varied across studies, with some focusing on children (ages 9–14) while others focused on adolescents and young adults (ages 15–26). All seven studies used the HPV vaccination rate as the primary outcome measure.

#### 3.1.1. Characteristics of Interventions

The interventions implemented in these studies shared the commonality of using EMRs; however, they differed in the specifics of their usage and supplementary interventions. In Dixon’s study (2019), the EMR system was supplemented by a mobile application [21]. Fiks (2013) coupled an EMR system with an automated educational program for parents, reinforcing parental awareness and commitment to timely vaccination [22]. Harry (2022) utilized a Clinical Decision Support System (CDSS) integrated within EMR, demonstrating an attempt to leverage decision-making algorithms to boost vaccination rates [23]. Similarly, Zimet et al. (2018) employed a CDSS, the Child Health Improvement through Computer Automation (CHICA) system, and automated reminders for healthcare providers to encourage HPV vaccination [24]. Szilagyi (2015) capitalized on the EMR system by establishing a reminder system within it [25]. Tiro (2015) adopted a more human-centered approach, augmenting the use of the EMR system with education for physicians and reminders for parents. This multicomponent intervention recognized the importance of provider and parent roles in the vaccination process [26]. Finally, Wilkinson (2019) utilized a computer-based intervention incorporating provider prompts into an EMR system. This method ensured that healthcare providers were regularly reminded of vaccination schedules during patient encounters [27].

#### 3.1.2. Additional Strategies: Provider Feedback

Fiks (2013) used provider feedback as an intervention. This involved a comprehensive system for tracking the vaccination status, setting reminders, and providing regular feedback to healthcare providers about their vaccination rates, ultimately improving the consistency and timeliness of vaccinations [22].

#### 3.1.3. Additional Strategies: Parental Education or Reminder

Parental education and reminders were employed as strategies in three of the studies [21,22,26]. These interventions involved informing parents about the importance of vaccinations [21,26] or providing reminders of scheduled vaccination appointments [22]. This strategy increases parents’ knowledge and awareness, encouraging them to vaccinate their children on time.

### 3.2. Meta-Analysis

All seven studies were included in the meta-analysis. Three outcomes were examined: (1) improvement in the HPV vaccination initiation rate; (2) improvement in the HPV vaccination completion rate; and (3) improvement in the percentage of patients receiving the next required HPV vaccine dose. Based on a prior systematic review of HPV vaccination improvement interventions, we assumed that the heterogeneity of the intervention effects across studies were large; therefore, we applied a random-effects model to estimate the pooled effect sizes.

#### 3.2.1. Improvements in HPV Vaccine Initiation Rates

**Figure 2:**
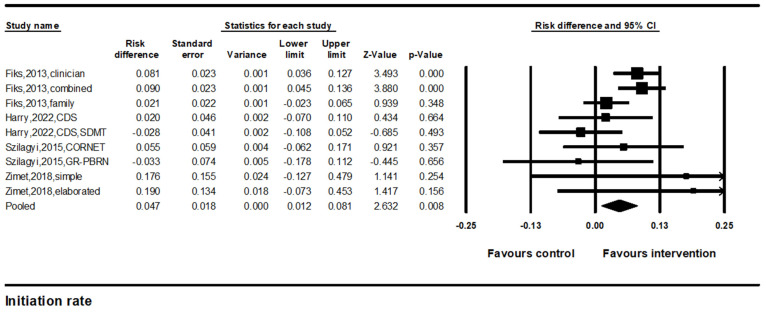
Forest plot of the effects on HPV vaccine initiation. Note. Forest plot showing the relative effect estimates for HPV vaccine initiation rates. The size of each square represents the weight of the study, and the horizontal line through the square indicates the 95% confidence interval (CI). The diamond represents the pooled estimate. Statistical heterogeneity: Q = 14.3, df(Q) = 8, *p* = 0.07, I^2^ = 40.8% [22,23,24,25].

Four studies with nine comparisons were included in the meta-analysis to estimate the pooled effects of improvements in HPV vaccine initiation rates. The results of the random-effects meta-analysis indicated a significant difference in the HPV vaccine initiation rate (pooled effect size = 4.7%, 95% confidence interval (CI): 1.2–8.1%, *p* < 0.01) (Figure 2). The results were consistent across studies (I^2^ = 40.8%).

#### 3.2.2. Improvements in HPV Vaccination Completion Rates

**Figure 3:**
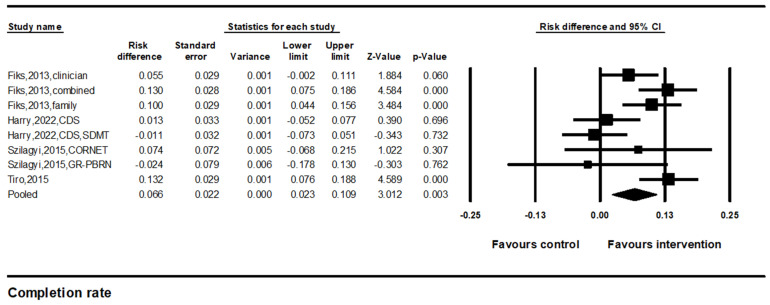
Forest plot of the effects on HPV vaccine completion. Note. Forest plot showing the relative effect estimates for HPV vaccine completion rates. The size of each square represents the weight of the study, and the horizontal line through the square indicates the 95% confidence interval (CI). The diamond represents the pooled estimate. Statistical heterogeneity: Q = 21.6, df(Q) = 7, *p* = 0.01, I^2^ = 67.3% [22,23,25,26].

We identified four studies with eight comparisons that compared the HPV vaccine completion rates between the intervention and control groups. The results of the random-effect meta-analysis indicated a significant difference in the HPV vaccine initiation rate (pooled effect size = 6.6%, 95% CI: 2.3–10.9%, *p* < 0.01) (Figure 3). However, the results were inconsistent across studies (I^2^ = 67.3%).

#### 3.2.3. Improvements in Receipt of the Next Needed Dose

**Figure 4:**
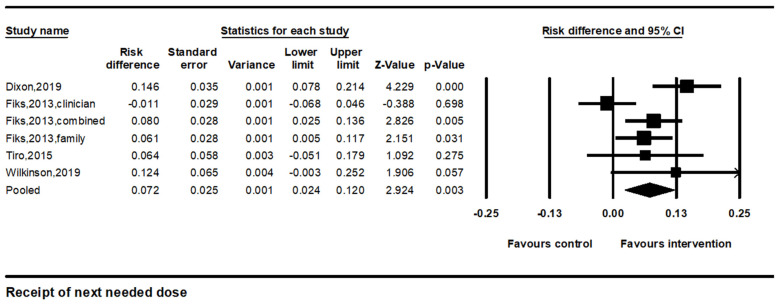
Forest plot of the effects on HPV vaccine receipt of the next needed dose. Note. Forest plot showing the relative effect estimates for receipt of the next HPV vaccine dose. The size of each square represents the weight of the study, and the horizontal line through the square indicates the 95% confidence interval (CI). The diamond represents the pooled estimate. Statistical heterogeneity: Q = 13.6, df(Q) = 5, *p* = 0.02, I^2^ = 63.2% [21,22,26,27].

We identified four studies with six comparisons that measured improvements in the percentage of patients who received their next vaccine dose. The percentage of those receiving the next HPV vaccine dose was defined as the number of eligible adolescents who received a subsequent dose. The pooled effect size was 7.2% (95% CI: 2.4–12.0%, *p* < 0.01). I^2^ was 63.2%, indicating considerable heterogeneity (Figure 4).

### 3.3. Subgroup Analysis

The subgroup analysis focused on the differential effects of provider feedback and parental education or reminders as supplementary to EMR-based interventions on the three HPV vaccination outcomes: initiation rate, completion rate, and receipt of the next needed dose (Table 2).

#### 3.3.1. Initiation Rates

Provider feedback differed significantly between EMRs-based reminders alone and reminders with provider feedback (Q (df = 1) = 8.60, *p* = 0.003). The intervention incorporating provider feedback was more effective, with an 8.6% increase (95% CI: 5.3–11.8%, *p* < 0.001), while reminders alone exhibited a non-significant increase of 1.7% (95% CI: −1.5–5.0%, *p* = 0.30). Parental education and reminders revealed no significant differences (Q (df = 1) = 0.14, *p* = 0.71).

#### 3.3.2. Completion Rates

Provider feedback revealed no significant difference between EMR-based reminders alone and reminders with feedback (Q (df = 1) = 0.67, *p* = 0.41). Parental education and reminders revealed significant differences (Q (df = 1) = 17.3, *p* < 0.001). Interventions that used both provider and parental strategies were more effective, with a 12.1% increase (95% CI: 8.8–15.3%, *p* < 0.001) compared with the provider alone strategy, which had a non-significant increase of 2.2% (95% CI: −1.1–5.5%, *p* = 0.19).

#### 3.3.3. Receipt of the Next Needed Dose

Provider feedback revealed no significant difference (Q (df = 1) = 1.43, *p*= 0.23). However, reminders alone were more effective, with a 9.7% increase (95% CI: 4.9–14.4%, *p* < 0.001), compared to reminders with feedback, which revealed a non-significant increase of 3.5% (95% CI: −5.5–12.4%, *p* = 0.45). Parental education and reminders revealed no significant differences (Q (df = 1) = 0.42, *p* = 0.52). However, interventions incorporating both provider and parental strategies were more effective, with an 8.9% increase (95% CI: 5.0%–12.7%, *p* < 0.001) compared with the provider alone strategy, which revealed a non-significant increase of 4.4% (95% CI: −8.6–17.4%, *p* = 0.51).

The analysis also examined the effect of integrating audit and feedback mechanisms within the interventions. The inclusion of these components was associated with a notable increase in vaccination uptake of 9.4% (95% CI: 3.0–15.9%, *p* = 0.001), although this was accompanied by high heterogeneity (I^2^ = 82%). In contrast, interventions lacking audit and feedback mechanisms showed a lesser effect of 2.4% (95% CI: 0.8–3.9%, *p* = 0.004), with moderate heterogeneity (I^2^ = 42%). The impact of audit and feedback was significant (Q = 4.107, df = 1, *p* = 0.043).

The most substantial effects were observed with the combined strategy of presumptive communication alongside audit and feedback, which yielded an increase in vaccination rates of 11.4% (95% CI: 8.0–14.8%, *p* < 0.001), with the lowest heterogeneity among the analyzed groups (I^2^ = 22%). Strategies that did not incorporate both elements had a significantly smaller effect size of 2.5% (95% CI: 1.1–3.9%, *p* < 0.001), with low heterogeneity (I^2^ = 24%). The advantage of the combined strategy was marked and statistically significant (Q = 14.095, df = 1, *p* < 0.001).

### 3.4. Publication Bias

**Figure 5:**
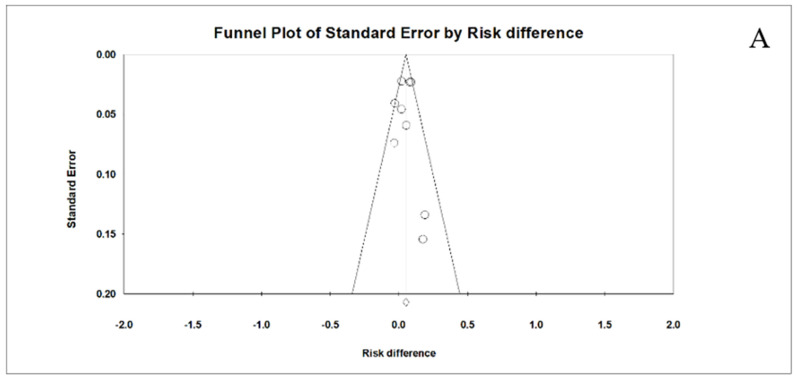

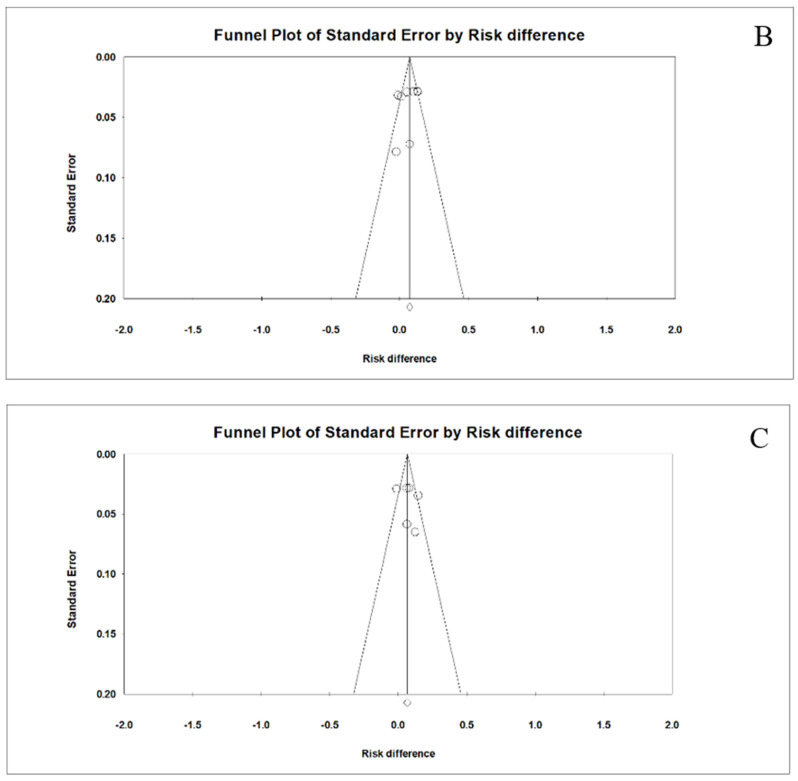
Funnel plot with symmetrical spread of effect sizes around the mean effect size for initiation rate (**A**), completion rate (**B**), and receipt of the next needed dose (**C**).

Visual inspection of the funnel plot showed no evidence for publication bias for all 3 outcomes: initiation rate (Figure 5A), completion rate (Figure 5B), and receipt of the next needed dose (Figure 5C). Distribution of effect sizes was fairly symmetrical. Most effect sizes fell in the funnel; while effect sizes falling outside the funnel did so symmetrically.

### 3.5. Risk of Bias

**Figure 6:**
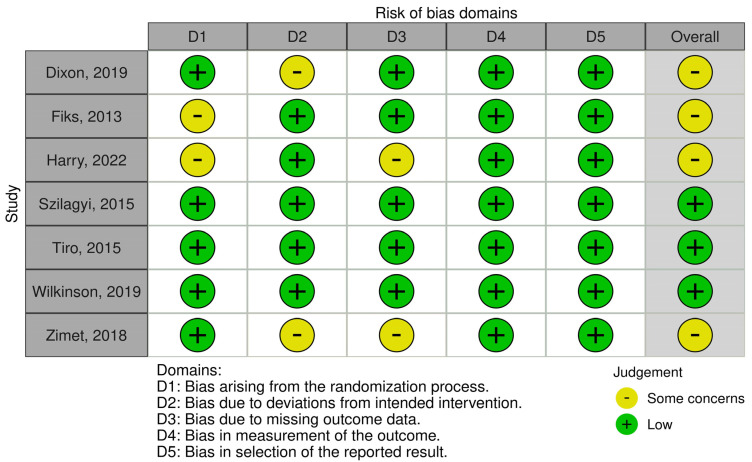
Risk of bias plot per study. Note. The methodological quality of the seven included studies assessed using the ROB 2.0 tool [21,22,23,24,25,26,27].

Figure 6 presents the methodological quality of the seven included studies. Overall, three studies satisfied all ROB 2.0 criteria and were deemed low risk for all five domains. More than half of the studies were judged as having some concerns as there were issues with the randomization process and deviations from the intended intervention. Two studies lacked information regarding the blinding of participants and interventionists. Due to the nature of certain interventions, it was not possible to blind interventionists. Such occurrences in clinical trials were viewed as contextual deviations that were unlikely to affect trial outcomes. The ROB 2.0 algorithm determined that these trials posed a low risk of bias.

## 4. Discussion

This systematic review and meta-analysis, based on seven important RCTs, emphasized the meaningful impact of EMR-based interventions on HPV vaccination uptake. Our in-depth analysis offers robust evidence for significant improvements in key areas: an average of 4.7% more patients initiated their HPV vaccination, an additional 6.6% of patients completed the HPV vaccine series, and 7.2% more patients received their next HPV vaccine dose on time. These practical and significant increases affirm the need for the wider implementation of EMR-based strategies in health systems to enhance HPV vaccination rates. While our analysis shows a statistically significant increase in HPV vaccination rates of 4–7% due to EMR-based interventions, it is important to note that this improvement may still be below the desired vaccination coverage levels of above 50%. This minimal percentage increase highlights the need for additional strategies to achieve higher vaccination rates.

Our study further expands on the meta-analysis of provider-based interventions by Wu et al. (2023) [13] by focusing on the use of EMR to promote HPV vaccination. This refined focus enables a deeper understanding of how technology can be leveraged in provider-based interventions to significantly improve HPV vaccination rates. While Wu et al. (2023) observed significant improvements in HPV vaccine initiation and the rate of patients receiving their next required dose, their study revealed no noticeable improvement in HPV vaccine completion rates through provider-based interventions. Our study fills this important gap. Our meta-analysis, emphasizing EMR-based interventions, reveals a significant enhancement in HPV vaccine initiation, receipt of the next required dose, and completion of the HPV vaccine series. The delineation of the role of EMR within provider-based interventions, as our study provides, is a crucial contribution to the field. This further underscores the potential of EMR to enhance the effectiveness of provider-based interventions and achieve holistic improvements in HPV vaccination rates.

EMR systems can automate the process of identifying and alerting providers about patients who are due for vaccination, thereby reducing the likelihood of missed vaccination opportunities. This is in line with a previous study, which suggested that using EMR reminders can effectively amplify vaccination rates by improving clinical workflow efficiency and alleviating the burden on providers. Moreover, coupling EMR-based provider reminders with patient or family reminders delivered via electronic channels can enhance patient engagement and promote shared decision-making [28,29,30]. This supports the study by Choi et al. (2023), which underscored the value of digital communication in reinforcing health education and behavioral nudges, thereby promoting acceptance and adherence to the HPV vaccine [31]. Our findings hold further significance as this systematic review and meta-analysis is the first to analyze the impact of EMR-based interventions on improving vaccination uptake in general and not limited to the HPV vaccine. This novelty extends the relevance of our results beyond HPV vaccination, substantially contributing to the broader field of vaccine promotion. By demonstrating how EMR can streamline vaccination practices and augment their efficiency, our study necessitates further exploration of the role of technology in enhancing public health initiatives. Ensuring vaccine uptake is more critical than ever; thus, the insights offered by our study can inform and improve vaccination strategies on a larger scale.

Our subgroup analysis, which analyzed the additive effects of provider feedback and parental education or reminders of EMR-based interventions on HPV vaccination outcomes, underscores the value of a multipronged approach. From our findings, EMR-based interventions supplemented with provider feedback significantly improved HPV vaccination initiation rates by 8.6% compared with reminders alone. This observation aligns with previous studies [32,33] that have highlighted the positive impact of provider feedback on improving vaccine uptake. These findings reinforce the notion that regular feedback can equip providers with key insights for modifying their approaches, leading to improved vaccination rates.

Regarding completion rates, combining provider feedback with parental education or reminders resulted in significant improvements in effectiveness, increasing the rates by 12.1%. This underscores the pivotal role of parental engagement in driving vaccination completion rates, a concept supported by a previous meta-analysis on the impact of parental reminders on immunization rates [34]. The consistent positive impact of the combined provider and parental strategies underscores the importance of multifaceted interventions. The combined use of technology and targeted education or reminders to engage providers and parents can create a conducive environment for improving HPV vaccination rates.

Our study had some limitations that should be considered. First, all the included studies were conducted in the United States, which might limit the generalizability of our findings to other healthcare systems or countries with different cultural, economic, and demographic characteristics. Second, although our analysis included a comprehensive selection of EMR-based interventions, other potential interventions that were not included in this review may have different effects. In addition, the included studies varied considerably in terms of methodological quality, which may have introduced bias into our pooled estimates. Moreover, while we controlled for some variations using a random-effects model, heterogeneity in the sample size, population, and intervention specificity may have influenced our results. Finally, although our study highlighted the beneficial role of combined provider- and client-based interventions, we did not dissect the independent contribution of the client-based component, which would be insightful for designing effective interventions.

The findings of this study have several key implications for clinical practice. The substantial improvements in HPV vaccination outcomes underscore the potential of EMR-based interventions, particularly when complemented by provider feedback and parental education or reminders. Clinics and health systems should consider incorporating these strategies into their workflow, given their demonstrated effectiveness in improving vaccination rates. Automated reminders and feedback systems through EMRs streamline the clinical workflow and provide real-time insights, facilitating more proactive management of patient vaccination schedules. Engaging parents through education or reminders provides additional support and increases the likelihood of completing the vaccination series.

Despite these promising findings, several areas warrant further investigation. Future studies should explore the differential effects of EMR-based interventions across various healthcare systems and countries with diverse cultural, economic, and demographic characteristics to enhance the generalizability of the findings. Research should also focus on identifying and addressing disparities in vaccine uptake among different sociodemo-graphic groups. Investigating the cost-effectiveness of these strategies is crucial for aiding health systems in making informed decisions regarding resource allocation. Additionally, research should examine how these strategies affect the uptake of vaccines other than HPV to determine their broader applicability. With rapid advancements in digital health technologies, future studies should explore new ways of leveraging these tools to optimize vaccination practices. This includes exploring the role of predictive analytics in identifying patients at risk of noncompliance and using patient portals to enhance patient–provider communication regarding vaccinations. Finally, research should examine the long-term sustainability of these interventions and their impact on overall population health outcomes. Understanding the independent contribution of client-based components within combined interventions would also provide valuable insights for designing effective strategies.

## 5. Conclusions

Our study underscores the substantial impact of EMR-based interventions on improving HVU, demonstrating considerable increases in the initiation, completion, and receipt of the next needed vaccine doses. As the first meta-analysis to broadly investigate the influence of EMR systems on vaccination practices, our findings advocate for more widespread integration of such digital health technologies in healthcare systems. By leveraging EMR strategies, we can bolster vaccination programs and contribute to wider disease prevention and public health objectives.

## Figures and Tables

**Table 1:** Basic characteristics across included studies.

Study	N	Child Age	Intervention	Control	Outcomes
Dixon 2019 [21]	1596 I = 2 clinics with 537 patients C = 3 clinics with 1059 patients	11–12 years	Automated medical assistant reminders in the electronic medical record (EMR) system with client education on tablets before seeing a physician	Usual care	Receipt of next needed vaccine dose
Fiks 2013 [22]	22,486 I1 = 5680 patients I2 = 5557 patients I3 = 5561 patients C = 5688 patients	11–17 years	I1: Family-focused intervention: Automated educational reminder calls to patients I2: Clinician-focused intervention: EMR-based vaccine alerts for clinicians; automated educational reminder calls to patients; performance feedback reports on vaccine delivery for clinicians I3: combined interventions I1 and I2	Standard care with no EMR-based alerts for adolescent vaccines, no education, and no feedback on adolescent vaccination rates	Initiation, Completion, Receipt of next needed vaccine dose
Harry 2022 [23]	6274 I1 = 11 clinics with 1897 patients I2 = 11 clinics with 1813 patients C = 12 clinics with 2564 patients	18–26 years	I1: Clinical Decision Support (CDS) system providing cancer prevention recommendations, including HPV vaccination I2: CDS system along with Shared Decision Making Tools for HPV vaccination	Usual care	Initiation, Completion
Zimet 2018 [24]	648 I1 = 8 physicians with 124 patients I2 = 11 physicians with 223 patients C = 10 physicians with 301 patients	11–13 years	CHICA system with automated provider reminders I1 = Simple reminder prompt I2 = Elaborated reminder prompt, which included suggested language for recommending the early adolescent platform vaccines	Usual practice control	Initiation
Szilagyi 2015 [25]	7040 I = 11 clinics: five local practices (GR-PBRN) with 800 patients and six national setting practices (CORNET) with 960 patients C = 11 clinics: five local practices (GR-PBRN) with 800 patients and six national setting practices (CORNET) with 960 patients	11–18 years	Provider prompts delivered either by nurse/staff during patient visits or via EMR. Monthly follow-up calls were also conducted with intervention practices	Usual care (no prompts)	Initiation, Completion
Tiro 2015 [26]	814 I = 410 patients C = 404 patients	11–18 years	Safety-Net clinic utilized EMR to identify the target population, monitor HPV vaccine status, obtain patient information, and assess outcomes. Mailing of educational materials, intervention components 2 and 3 (recalls), and delivery of recalls for each dose	Active Comparison group received a CDC brochure about all Advisory Committee on Immunization Practices recommended vaccines. No active contact or EHR utilization in this group	Completion, Receipt of next needed vaccine dose
Wilkinson 2019 [27]	1285 I = 15 physicians with 634 patients C = 14 physicians with 651 patients	11–17 years	CHICA system to automatically check immunization records, verify patient eligibility, and prompt physicians to order the second and third doses of HPV vaccine during eligible patient encounters	Usual practice where nurses manually obtain vaccination recommendations	Receipt of next needed vaccine dose

I, Intervention; C, Control; GR-PBRN, Greater Rochester Practice-Based Research Network; CORNET, The Continuity Research Network; CHICA, the Child Health Improvement through Computer Automation.

**Table 2:** Additional strategies associated with intervention effectiveness in HPV vaccination outcomes.

Vaccination Outcomes	EMR-Based Intervention Modalities	Relative Effect Estimate (95% CI)	*p*-Value	No. of Effect Sizes	I^2^
Initiation Rates	1. Provider feedback	Q (df = 1) = 8.60	0.003		
	Reminder alone	1.7% (−1.5–5.0%)	0.30	7	0
	Reminder plus feedback	8.6% (5.3–11.8%)	<0.001	2	0
	2. Parental education or reminder	Q (df = 1) = 0.14	0.71		
	Provider alone	4.0% (−0.9–8.8%)	0.11	7	32
	Provider plus parental education or reminder	5.5% (−1.3–12.3%)	0.11	2	78
Completion Rates	1. Provider feedback	Q (df = 1) = 0.67	.041		
	Reminder alone	5.4% (−0.2–11.0%)	0.06	6	69
	Reminder plus feedback	9.3% (1.8–16.7%)	0.01	2	71
	2. Parental education or reminder	Q (df = 1) = 17.3	<0.001		
	Provider alone	2.2% (−1.1–5.5%)	0.19	5	0
	Provider plus parental education or reminder	12.1% (8.8–15.3%)	<.001	3	0
Receipt of the Next Needed Dose	1. Provider feedback	Q (df = 1) = 1.43	0.23		
	Reminder alone	9.7% (4.9–14.4%)	<0.001	4	26
	Reminder plus feedback	3.5% (−5.5–12.4%)	0.45	2	80
	2. Parental education or reminder	Q (df = 1) = 0.42	0.52		
	Provider alone	4.4% (−8.6–17.4%)	0.51	2	72
	Provider plus parental education or reminder	8.9% (5.0–12.7%)	<0.001	4	24

## Data Availability

The data supporting the findings of this systematic review and meta-analysis are derived from publicly available articles. Detailed references to the original studies are provided within the manuscript. Data generated during the analysis are contained within this published article. For access to these data, inquiries can be directed to the corresponding author, who will facilitate data requests as per applicable privacy and ethical guidelines.
